# Safety and outcome of elective synthetic mesh repair for incisional ventral hernias in immunosuppressed patients – a retrospective propensity-score-matched analysis

**DOI:** 10.1007/s10029-025-03273-3

**Published:** 2025-02-24

**Authors:** Ramin Raul Ossami Saidy, Yvonne May Callister, Luca Dittrich, Dennis Eurich, Christian Denecke, Johann Pratschke, Jonas Raakow

**Affiliations:** Department of Surgery, Charité Universitätsmedizin Berlin, Berlin, Germany

**Keywords:** Incisional ventral hernia, Mesh repair, Immunosuppression

## Abstract

**Introduction:**

Incisional hernia remain an important complication after abdominal surgery. Repair often includes use of synthetic mesh, but certain risk factors for complication after mesh hernia repair have been described. Among these, immunosuppression due to co-existing conditions is hypothesized to increase postoperative complications, but data is scarce and contradicting. Therefore, the aim was to assess outcome after mesh hernia repair in immunosuppressed patients.

**Material & methods:**

Patients with and without immunosuppression undergoing elective incisional hernia repair at our clinic between 2010 and 2019 were analyzed in this retrospective study. Pre-existing conditions, details of immunosuppression, postoperative course and outpatient follow-up for hernia recurrence were collected and impact of clinical variables on outcome was analyzed. Propensity score matching was performed for comparison of cohorts.

**Results:**

Immunosuppression was associated with increased postoperative complications in the overall cohort of 732 patients undergoing incisional ventral hernia repair in univariate but not multivariate analysis (p = 0.036 and p = 0.25, respectively). Overall postoperative complications did not differ between patients with immunosuppression compared to the matched collective. However, use of > 2 immune suppressive agents and immunosuppression history > 48 months showed significant impact on postoperative complications in univariate and multivariate analysis (p = 0.003/p = 0.023 and p = 0.018/p = 0.03, respectively). Age (< 60 years), duration of surgery (> 120 min), midline hernia according to EHS classification and number of immunosuppressive agents administered were identified as important risk factors for recurrence in immunocompromised patients (p = 0.045, p = 0.023, p = 0.012 and 0.049, respectively).

**Conclusion:**

In this study, overall safety with desirable outcome of mesh implantation in immunosuppressed patients was documented. Furthermore, data suggested significant impact of number of immunosuppressive agents as a predicator of postoperative complications in this collective, possibly enabling risk stratification within this subgroup.

**Supplementary Information:**

The online version contains supplementary material available at 10.1007/s10029-025-03273-3.

## Introduction

Incisional herniae are a common clinical problem and one of the most frequent long-term complications after abdominal operations with a reported incidence of up to 22.4% three years after midline laparotomy [1]. The incidence increases to more than 30% after midline laparotomy when patients are overweight with a body mass index (BMI) exceeding 27 kg/m² [2]. Also, recent chemotherapy, surgical site infection (SSI), active nicotine consumption, immunosuppression and end colostomy are also associated with increased hernia occurrence [3–6].

The current standard of treatment is surgical hernia repair including the use of permanent synthetic mesh as reinforcement of the abdominal wall [7]. This method can reduce the risk of recurrent hernias, but may cause serious clinical problems, if SSI occurs [8]. According to the Ventral Hernia Working Group, recurrence and SSI are the main issues in ventral hernia repair [9]. Recurrence rates are described to be up to 20–25% within three years following ventral hernia repair using synthetic mesh [10, 11]. The occurrence of SSI has been observed as a major risk factor for hernia recurrence, that may increase the rate up to 80% [3, 10].

Prevalence and incidence of patients classified as “immunosuppressed” is likely to increase due to expanding therapeutical options for and a rising population of patients undergoing administration of immunosuppressive agents in autoimmunological, rheumatological and oncological diseases as well as organ transplantation. However, a systematic classification is not available [12–14]. Simultaneously, immunosuppression is linked to postoperative complications, such as wound healing disorders and SSI, that may lead to mesh resection [15–18]. The Ventral Hernia Working Group classifies immunosuppressed patients into “Grade 2 - comorbid” in their assessment of risk of SSI, implying increased risk of infection of synthetic material [9]. An analysis of the Americas Hernia Society Quality Collaborative observed more surgical site occurrences but not infections in immunocompromised patients [19]. Further, immunosuppression has been identified as a risk factor for hernia recurrence [20]. A meta-analysis reported 13.5% surgical complications after mesh hernia repair in patients after renal transplantation, while another study found no differences in complications between patients undergoing incisional hernia repair after liver transplantation compared to a control group [21, 22].

This study aimed to determine postoperative outcome of synthetic mesh implantation for ventral hernia repair in immunosuppressed patients in regard to postoperative complications as well as hernia recurrence with focus on the impact of extent of immunosuppression.

## Materials and methods

### Cohort and inclusion criteria

All patients undergoing ventral hernia repair for incisional herniae between January 2010 and December 2019 at Surgical Department of the Charité – Universitätsmedizin Berlin were included in the analysis. Immunosuppressed patients were defined by (i) taking immunosuppressive drugs with systemic effect or (ii) by the definition of their disease (e.g. Human immunodeficiency virus, HIV). Patients undergoing topical steroid therapy without systemic effects were not classified as immunosuppressed. Patients with oncological disease were only considered immunosuppressed if systemic therapy was administered within four weeks prior to surgery.

Patients were excluded from the analysis if the hernia repair was performed as part of a more complex operation (e.g. (oncological) resection, ostomy reversal surgery, organ (transplant) removal) or emergency cases with signs of incarceration. Patients undergoing sole suture repair were also excluded from the analysis.

Herniae were mainly diagnosed by general practitioners and then referred to our outpatient clinic, where diagnosis was confirmed by physical examination and ultrasound and/or computed tomography. Surgery was performed in symptomatic patients only.

### Clinical data and follow-up

A follow-up via telephone was conducted at 3, 6, 12, 24 and 36 months after surgery by a hernia surgeon for the cohort of immunosuppressed patients. The main objective was to determine rate of recurrent hernias. Follow-up time was determined as time from hernia repair until further abdominal surgery or date of call. Standardized questions using patient-reported outcomes (PROs) as described by Baucom et al. were asked in reference to the follow-up time period: patients were asked (i) if they had experienced symptoms like pain, pressure or discomfort in the area of hernia repair and if (ii) bulging reoccurred [23]. Patients reporting recurrent bulging and/or persistent pain at the original repair site were offered to be examined by a surgeon. Patients declining symptoms were categorized without hernia recurrence.

Data for pre-existing conditions were collected from patients` anamnesis. Diabetes was recorded, if any pharmaceutical therapy was administered.

Further data, including surgical complications were collected from hospital stays, ambulatory visits of the patients in the transplant centers or other contacts with surgeons and physicians at Charité – Universitätsmedizin Berlin. For analysis, surgical complication was recorded, if either of the following occurred in the postoperative course: wound healing disorders, paralytic ileus and events directly linked to surgical intervention (bleeding, injuries). Cardiopulmonary complications included arrythmia with need of observation/treatment in an intensive care unit, prolonged necessity of (non-)invasive ventilation, pulmonary or cardiac embolism as well as pneumonia. Renal complications included acute kidney injury or pyelonephritis. Further, non-surgical site infection (e.g. urinary tract infections), post-operative delirium or other occurrences were recorded. The Clavien-Dindo-Classification was used to categorize surgical complications [24].

### Statistical analysis

Data were collected in a prospectively designed database and a propensity score-matching was conducted using the following variables: age, BMI, pre-existing diabetes or nicotine consumption, anticoagulation, risk classification according to the American Society of Anesthesiologists (ASA–score), recurrent hernia, classification of incisional abdominal wall herniae according to the European Hernia Society (EHS) and surgical approach (laparoscopic vs. open) [25, 26]. A match tolerance of 0.1 was acknowledged.

To enable comparative group analysis, following parameters were dichotomized: age (≤ 59.9 vs. > 60 years), BMI (≤ 29.9 vs. > 30 kg/m²) as well as duration of operation (≤ 120 vs. > 120 min) [27, 28].

Classification and regression tree (CART) analysis using Chi-squared Automatic Interaction Detection approach was conducted to evaluate impact of parameters on complications after hernia repair. Minimum of cases for upper and lower nodes were defined as 50 and 25, respectively.

All analyses were performed using Statistical Product and Service Solutions (SPSS), version 29 (IBM, USA). Variables are described as numbers with percentages as appropriate, or as means ± standard deviation (SD). The Chi square test (χ^2^) was used for analysis of categorical variables, and Student’s t test for numerical variables. Parameters, that revealed statistical significance in univariate analysis were included in multivariate analysis. Here, a logistic regression model, including demographic and operative parameters, was used to assess risk factors for postoperative complications. Hazard Ratio (HR) with Confidence interval (CI) were calculated. A p-value of < 0.05 was considered statistically significant.

The Strengthening the Reporting of Observational Studies in Epidemiology (STROBE) guidelines were used in this study´s design [29].

This study was conducted according to the guidelines of the Declaration of Helsinki and was approved by the local ethics committee of Charité Universitätsmedizin Berlin (EA1/067/20).

## Results

### Overall cohort characteristics and outcome

**Fig. 1:**
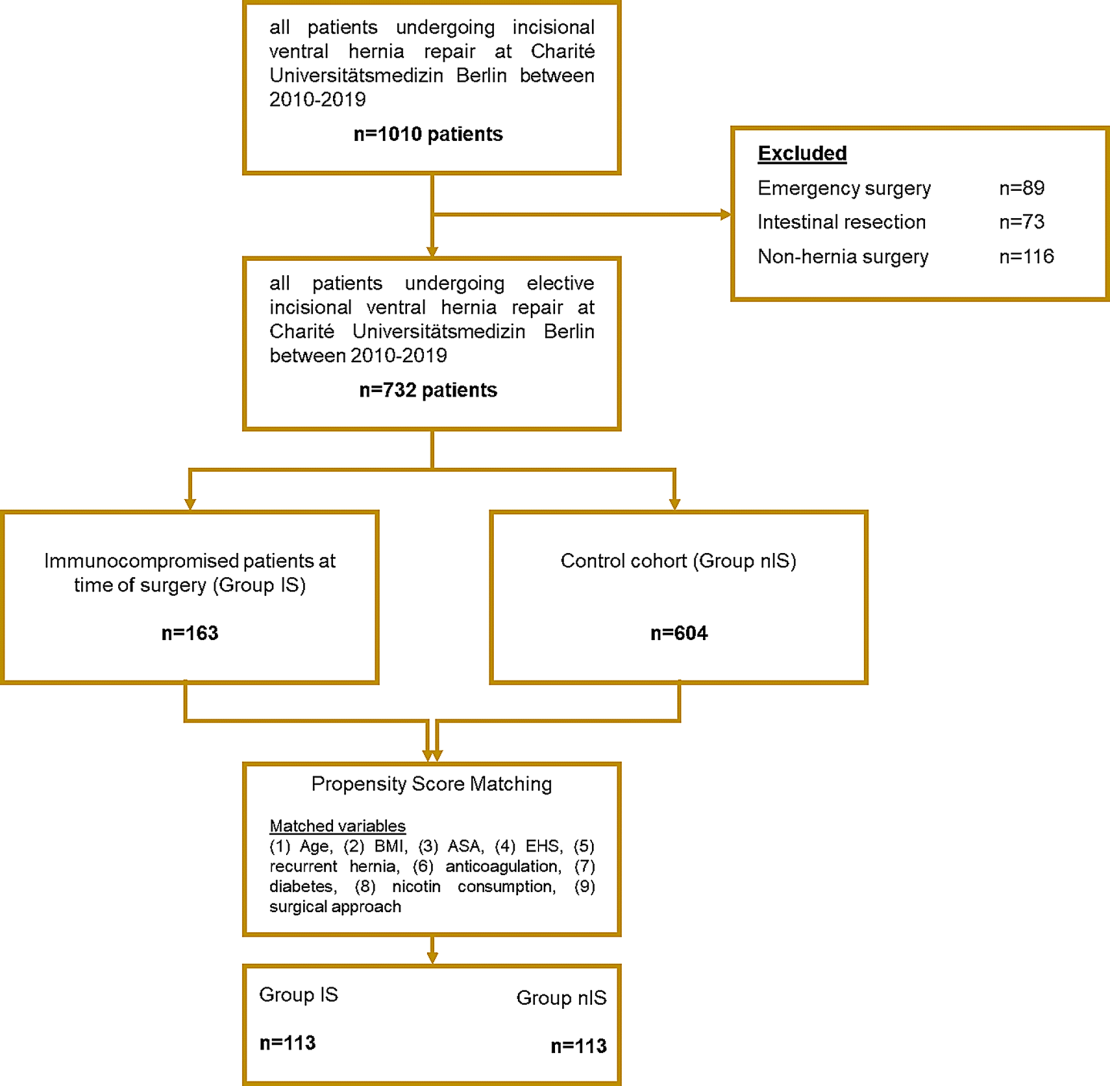
Formation of the Cohort and Subgroups. ASA - American Society of Anesthesiologists; BMI - Body Mass Index; EHS - European Hernia Society

Out of 732 patients that underwent elective surgery between 2010 and 2019 because of incisional abdominal wall hernia at our institution, a total of *n* = 163 (22.3%) patients were classified as immunosuppressed, see Fig. 1. The mean age of the overall cohort was 58.7 (± 12.5) years and most patients were classified as ASA 1 or 2 (*n* = 347; 47.4%). Using the EHS classification, the majority of herniae was classified as “midline” (*n* = 555;75.8%) and W2 or W3 (*n* = 650; 88.8%). 119 (16.3%) patients were operated due to recurrent hernia. Open hernia repair was conducted in 454 (62.0%) patients and sublay (*n* = 348; 47.5) was performed most frequently, followed by intraperitoneal onlay mesh (IPOM, *n* = 307; 41.0%).The mean duration of surgery was 149.3 (± 82.7) minutes. Detailed characteristics of the overall cohort are displayed in Table 1.

**Table 1:** Overall cohort characteristics

	Overall cohort *n* = 732	Patients with immunosuppression *n* = 163
Age in years;	58.7 ± 12.5	55.5 ± 10.9
BMI in kg/m^2,^	27.4 ± 7.8	26.6 ± 6.5
ASA		
1/2 3/4 Missing	347 (47.4) 251 (34.3) 134 (18.3)	53 (32.5) 82 (50.3) 28 (17.2)
COPD	75 (10.2)	16 (9.8)
Diabetes	132 (18.0)	52 (31.9)
Active nicotine consumption	112 (15.3)	13 (8.0)
Oncologic disease	267 (36.5)	46 (28.2)
Reason for IS		
Solid OT LT KT Other IBD Rheumatological HIV	-	150 (92.0) 98 (60.1) 37 (22.7) 15 (9.2) 4 (2.5) 6 (3.7) 3 (1.8)
Recurrent Hernia	119 (16.3)	24 (14.7)
Hernia Size in cm		
Length Width	9.9 ± 6.6 9.3 ± 6.3	11.2 ± 7.8 10.2 ± 7.1
EHS		
Midline (M1-5) Lateral (L1-4)	555 (75.8) 177 (24.2)	109 (66.9) 54 (33.1)
EHS		
W1 W2 W3	82 (11.2) 330 (45.1) 320 (43.7)	13 (8.0) 73 (44.8) 77 (47.2)
Surgical approach		
Open Laparoscopic	454 (62.0) 278 (38.0)	89 (54.6) 74 (45.4)
Type of Repair		
Onlay Sublay IPOM Other	40 (5.5) 348 (47.5) 307 (41.9) 37 (5.1)	9 (5.5) 69 (42.3) 80 (49.1) 5 (3.1)
Duration of surgery in minutes	149.3 ± 82.7	157.1 ± 74.1
Follow-up completion (%)	-	155 (95.1%)
Months after surgery ≥ 12 months ≥ 24 months ≥ 36 months	- - - -	26.8 (± 20.0) 120 (73.6) 70 (42.9) 52 (31.9)

Values as numbers and percentage or in means ± standard deviation

ASA - American Society of Anesthesiologists; BMI - Body Mass Index; COPD - Chronic Obstructive Pulmonary Disease; CNI - calcineurine inhibitor; EHS - European Hernia Society; IBD – inflammatory bowel disease, LT – liver transplantation; KT – kidney transplantation; OT – organ transplantation; IPOM – intraperitoneal onlay mesh

Postoperative complications occurred in 166 (22.7%) patients, and here, the majority was classified as Clavien-Dindo grade ≤ IIIa (*n* = 137; 18.7%). SSI were the most common surgical postoperative complication (*n* = 35; 4.8%). The mean duration of hospitalization was 8.6 (± 11.1) days, see also Table 2.

### Parameters associated with complications in the overall cohort

Univariate analysis revealed age (> 60 years) and patients classified as ASA 3/4 but not BMI as variables significantly associated with complications (*p* < 0.001, *p* < 0.001 and *p* = 0.49 respectively). Analyzing specific comorbidities, preexisting chronic obstructive pulmonary disease (COPD), diabetes and immunosuppression also showed significant impact on incidence of postoperative complications (*p* < 0.001, *p* = 0.04 and *p* = 0.036, respectively). Using EHS classification for ventral incision hernia, only larger width but not location reached statistically significant impact on complications (*p* = 0.003 and *p* = 0.99, respectively). Further, duration of surgery exceeding 120 min was associated with increased occurrence of postoperative complications (*p* < 0.001). Supplement Table 1 displays detailed information of the univariate analysis.

In multivariate analysis, only age > 60 years, COPD and duration of surgery > 120 min showed persistent statistically significant impact (*p* = 0.002, *p* = 0.032 and *p* < 0.001, respectively), see also Supplement Table 2.

**Fig. 2:**
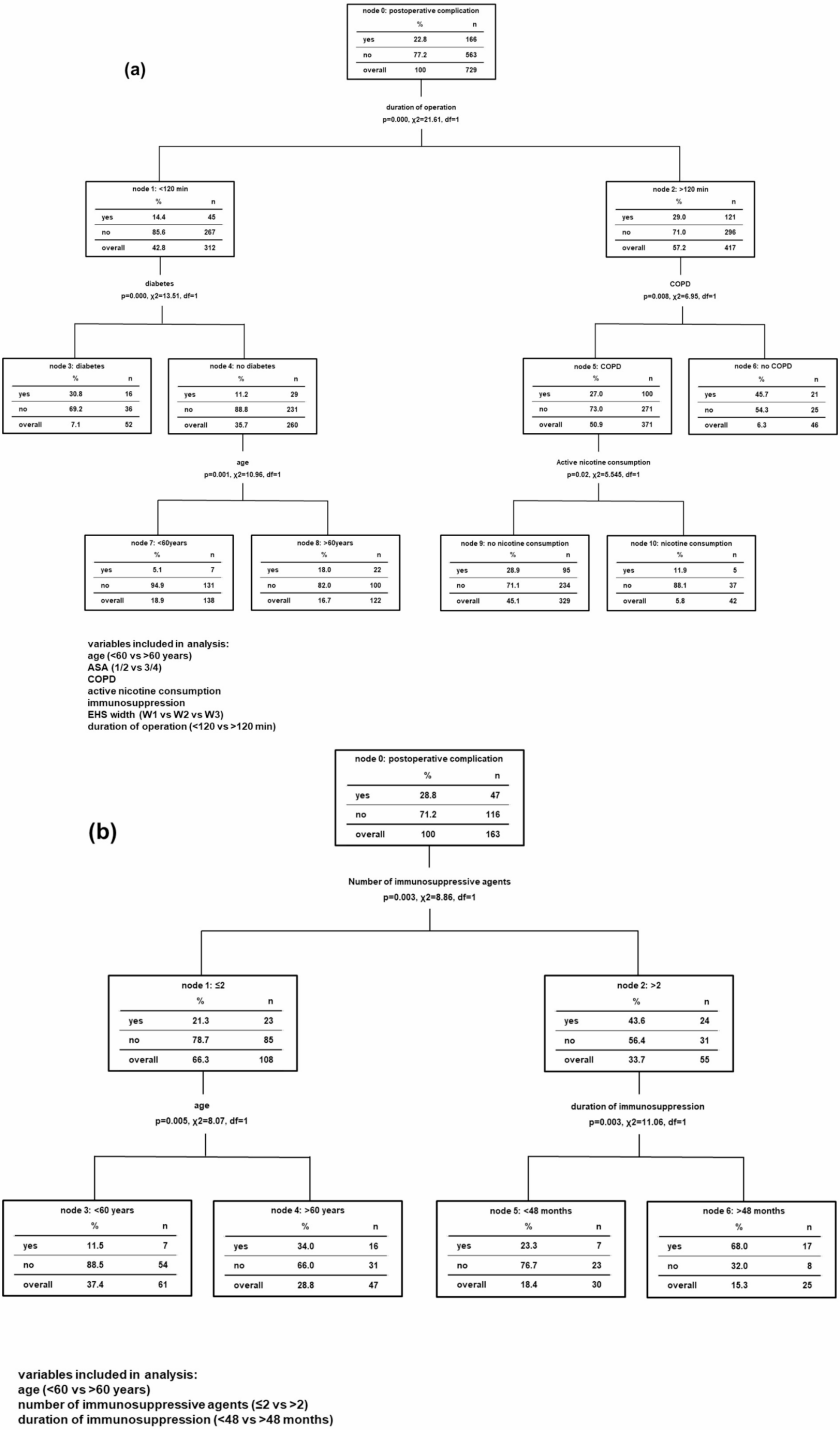
Classification and regression tree (CART)-analysis on impact of clinical variables on occurence of postoperative complications after incisional ventral hernia repair using mesh. To investigate and rank the relevance of clinical variables with putative influence on postoperative complications, CART method was used. Regression tree classified patients in this study for their associated variables in regard of occurrence of postoperative complications. Nodes indicate parameters that significantly improve correct classification and hereby decrease impurity toward the terminal (upper) nodes. Variables with statistically significant impact in univariate analysis were implemented. (**a**) In the overall cohort, duration of operation showed most relevant impact, and in the subgroup of patients operated < 120 min, diabetes mellitus was of importance, while combination of COPD and prolonged length of operation led to increase of postoperative complication. (**b**) In the cohort of immunosuppressed patients, the number of immunosuppressive agents was of most meaningful impact. Here, in patients with up to two agents, increased age was next-ranking factor, while history of immunosuppression exceeding four years was of relevance in patients with more than two different pharmaceuticals

CART analysis revealed duration of operation as most impactful variable for occurrence of postoperative complications in the overall cohort, followed by comorbidities. In the subset of patients where operation was under 120 min, diabetes was most relevant, while COPD showed most important influence in patients, where operation time was > 120 min. Immunosuppression did not show significant impact in this analysis, see also Fig. 2a.

### Characteristics and outcome of immunosuppressed patients

The mean age of patients classified as immunosuppressed was 55.5 (± 10.9) years and most patients were classified with higher ASA gradings of 3 and 4 (*n* = 82; 50.3%). Preexisting diabetes was reported in 52 (31.9%) of patients. Immunosuppression was mainly caused by pharmacological therapy due to rejection prophylaxis for solid organ transplantation (*n* = 150; 92.0%). Here, most patients were recorded with a medical history of liver transplantation (*n* = 98; 60.1%). Similar to the overall cohort, midline herniae (*n* = 109; 66.8%) with larger width of W2/W3 (*n* = 150; 92.0%) were most prominent. Laparoscopic approach was performed in 74 (45.4%) patients and hernia repair using IPOM technique was most frequent (*n* = 80; 49.1%), see also Table 1.

Overall, complications in this cohort were observed in 47 (28.8%) patients and here, SSI were diagnosed in 12 (7.4%) of patients. Complications exceeding Clavien-Dindo IIIa were found in 14 (8.6%) patients while mean hospitalization was 9.3 (± 10.7) days. Details on complications in immunosuppressed patients are displayed in Table 2.

**Table 2:** Early postoperative outcome

	Overall cohort *n* = 732	Patients with immunosuppression *n* = 163
Patients with postoperative complications	166 (22.7)	47 (28.8)
Patients with postoperative surgical complications	109 (14.9)	24 (14.7)
Typ of complications*		
SSI Seroma Bleeding/hematoma Paralytic Ileus Renal Cardiopulmonary Other	35 (4.8) 30 (4.1) 12 (1.6) 8 (1.1) 21 (2.9) 27 (3.7) 80 (10.9)	12 (7.4) 7 (4.3) 1 (0.6) 0 (0) 7 (4.3) 7 (4.3) 31 (19.0)
Grade of complication (Clavien-Dindo)		
≤IIIa ≥IIIb	137 (18.7) 29 (4.0)	33 (20.2) 14 (8.6)
Length of Hospital stay in days	8.6 ± 11.1	9.3 ± 10.7

*multiple selection possible

values as numbers and percentage or in means ± standard deviation

SSI – surgical site infection; CDC – Clavien-Dindo Classification

### Parameters associated with complications in immunosuppressed patients

In the cohort of immunosuppressed patients, only patients aged > 60 years, patients under therapy with more than two immunosuppressive agents and duration of immunosuppressive therapy beyond 48 months were associated with statistically significant increased rate of complications (*p* = 0.035, *p* = 0.003 and *p* = 0.018), see Table 3.

**Table 3:** Univariate analysis of impact of clinical variables associated with postoperative complications in immunosuppressed patients

variable	Patients with postoperative complications (%)	*p* value
Age		
< 60 years ≥ 60 years	22 (22.7) 25 (37.9)	**0.035**
BMI		
< 30 kg/m² > 30 kg/m²	32 (28.3) 13 (28.3)	0.994
ASA		
1/2 3/4	13 (24.5) 25 (30.5)	0.452
Comorbidities		
COPD Active nicotine consumption Diabetes Oncologic disease	7 (43.8) 5 (38.5) 19 (36.5) 12 (26.1)	0.165 0.424 0.137 0.627
EHS		
Midline (M1-5) Lateral (L1-4)	32 (29.4) 15 (27.8)	0.834
EHS		
W1 W2 W3	2 (15.4) 18 (24.7) 27 (35.1)	0.20
Surgical approach		
Open Laparoscopic	25 (28.1) 22 (29.7)	0.818
Duration of surgery		
< 120 min > 120 min	17 (27.0) 30 (30.0)	0.679
Reason for immunosuppression		
LT KT Other OT IBD Rheumatological HIV	28 (28.6) 13 (35.1) 3 (20.0) 3 (50.0) 0 (0) 0 (0)	0.365
Class of immunosuppressive agent		
CNI Steroids MMF Antibodies Other	44 (29.7) 19 (33.9) 17 (27.4) 3 (37.5) 8 (29.6)	0.428 0.299 0.755 0.579 0.920
Number of immunosuppressive agents		
≤ 2 > 2	23 (21.3) 24 (43.6)	**0.003**
Duration of immunosuppression		
> 12 months > 24 months > 36 months > 48 months	34 (28.1) 25 (31.3) 23 (35.9) 20 (40.8)	0.820 0.409 0.076 **0.018**

values as numbers and percentage

ASA - American Society of Anesthesiologists; BMI - Body Mass Index; COPD - Chronic Obstructive Pulmonary Disease; CNI - calcineurine inhibitor; EHS - European Hernia Society; MMF - Mycophenolat-Mofetil; IBD – inflammatory bowel disease, LT – liver transplantation; KT – kidney transplantation; OT – organ transplantation

Multivariate analysis implementing these parameters verified statistical significant association for “age” (HR 0.45, 95%CI 0.21–0.97, *p* = 0.042), “number of immunosuppressive agents” (HR 0.41, 95%CI 0.19–0.89, *p* = 0.023) and “duration of immunosuppression” (HR 0.42, 95%CI 0.19–0.92,*p* = 0.03), see Table 4.

**Table 4:** Multivariate logistic regression of parameters associated with postoperative complications in immunosuppressed patients undergoing incision hernia repair

Variable	*n*	Hazard Ratio	95% CI		*p* value
			lower	upper	
Age (< 60* vs. ≥60 years)	144	0.446	0.205	0.972	**0.042**
Number of immunosuppressive agents ≤ 2* vs. >2	144	0.406	0.186	0.885	**0.023**
Duration of immunosuppression ≤ 48* vs. > 48 months	144	0.422	0.193	0.922	**0.03**

*Reference

Values as numbers

CI - Confidence interval

CART analysis identified the number of immunosuppressive agents with most relevance regarding postoperative complications. Patients´ age was most impactful in the subset of patients with up to two immunosuppressive agents and duration of immunosuppression in patients with more than two immunosuppressant, see Fig. 2b.

### Parameters associated with hernia recurrence in immunosuppressed patients

Follow-up was available in 155 (95.1%) of patients and mean follow-up was 26.8 (± 20.0) months after hernia repair. During this period, 32 (19.6%) patients reported pain and/or bulging. All of these patients were eventually examined by a surgeon and recurrent hernia was observed in 23 (14.1%) patients, resulting in a specificity of PROs of 71.8%.

Univariate analysis revealed increased rate of recurrence in patients younger than 60 years and in midline herniae (*p* = 0.045 and *p* = 0.012, respectively). Further, laparoscopic approach and duration of operation > 120 min were found with statistically significant impact (*p* = 0.036 and *p* = 0.023, respectively). A statistically significant, twofold increase of recurrent herniae was observed in patients with more than two immunosuppressive agents (*p* = 0.049). For details see Table 5.

**Table 5:** Univariate analysis on impact of clinical parameters on recurrence of hernia in immunosuppressed patients

Variable	Recurrence in Patients with immunosuppression *n* = 163	*p* value
Age		
< 60 years ≥ 60 years	18 (19.6) 5 (7.9)	**0.045**
BMI		
< 30 kg/m² > 30 kg/m²	15 (14.2) 8 (17.8)	0.571
ASA		
1/2 3/4	9 (17.6) 11 (14.5)	0.630
Comorbidities		
COPD Active nicotine consumption Diabetes Oncologic disease	4 (25.0) 1 (7.7) 8 (15.7) 4 (9.3)	0.227 0.449 0.835 0.230
Recurrence after prior hernia reparation	4 (16.7)	0.813
EHS		
Midline (M1-5) Lateral (L1-4)	21 (19.6) 2 (4.2)	**0.012**
EHS		
W1 W2 W3	0 (0) 9 (13.2) 14 (18.9)	0.185
Surgical approach		
Open Laparoscopic	8 (9.4) 15 (21.4)	**0.036**
Duration of surgery		
< 120 min > 120 min	4 (6.7) 19 (20.0)	**0.023**
Reason for Immunosuppression		
LT KT Other OT IBD Rheumatological HIV	17 (18.3) 2 (5.9) 4 (26.7) 0 (0.0) 0 (0) 0 (0)	0.224
Class of Immunosuppressive Agent		
CNI Steroids MMF Antibodies Other	23 (16.4) 8 (15.1) 9 (15.5) 0 (0) 6 (24.0)	0.089 0.949 0.854 0.225 0.159
Number of Immunosuppressive Agents		
≤ 2 > 2	11 (10.8) 12 (22.6)	**0.049**
Duration of Immunosuppression		
> 12 months > 24 months > 36 months > 48 months	5 (23.8) 10 (16.7) 11 (14.9) 14 (15.7)	0.200 0.566 0.954 0.643

values as numbers and percentage

ASA - American Society of Anesthesiologists; BMI - Body Mass Index; COPD - Chronic Obstructive Pulmonary Disease; CNI - calcineurine inhibitor; EHS - European Hernia Society; MMF - Mycophenolat-Mofetil; IBD – inflammatory bowel disease, LT – liver transplantation; KT – kidney transplantation; OT – organ transplantation

### Outcome after ventral incisional hernia repair after propensity score matching

After propensity score matching using eight clinical variables, *n* = 113 patients with ongoing immunosuppression (group IS) were compared to *n* = 113 patients without (group nIS), see Fig. 1; Table 6. Overall complications did not differ between groups (*n* = 31/27.4% vs. *n* = 31/27.4%; *p* = 0.88). Surgical complications were significantly more frequent in the control group (nIS; *n* = 24/21.2% vs. IS; *n* = 15 (13.3%), *p* = 0.02). Conversely, increased renal complications were observed in the IS group (IS; *n* = 8/7.1% vs. nIS; *n* = 2/1.8%, *p* = 0.05). see Table 7. All patients with renal complications had undergone organ transplantation (liver transplantation: *n* = 4, kidney transplantation: *n* = 2, combined kidney/pancreas transplantation: *n* = 2).

**Table 6:** Group characteristics and univariate analysis after propensity score matching

	Immunosuppression (Group IS) *n* = 113	No Immunosuppression (Group nIS) *n* = 113	*p*-value
Age* in years;	57.1 ± 10.3	56.0 ± 13.7	0.49
BMI* in kg/m^2,^	26.6 ± 7.3	26.7 ± 10.4	0.89
ASA*			0.42
< 3 ≥ 3	71 (62.8) 42 (37.2)	65 (57.5) 48 (42.5)	
COPD	12 (8.0)	9 (10.6)	0.49
Diabetes*	32 (28.3)	25 (22.1)	0.28
Active nicotine consumption*	11 (9.7)	11 (9.7)	1.0
Oncologic disease	34 (30.1)	36 (31.9)	0.77
Reason for IS	101 (89.4)	-	-
Solid OT LT KT Other IBD Rheumatological HIV	66 (58.4) 25 (22.1) 10 (8.8) 4 (3.5) 5 (4.4) 3 (2.7)		
Recurrent Hernia*	17 (15.0)	17 (15.0)	1.000
Hernia Size in cm			
Length Width	10.9 ± 7.5 9.8 ± 6.2	9.5 ± 5.6 9.7 ± 6.3	0.13 0.90
EHS*			0.64
Midline (M1-5) Lateral (L1-4)	84 (74.3) 29 (25.7)	87 (77.0) 26 (23.0)	
EHS*			
W1 W2 W3	8 (7.1) 51 (45.1) 54 (47.8)	9 (8.0) 51 (45.1) 53 (46.9)	0.97
Surgical approach			
Open Laparoscopic	60 (53.1) 53 (46.9)	61 (54.0) 52 (46.0)	0.89
Type of Repair			
Onlay Sublay IPOM Other	4 (3.5) 46 (40.7) 60 (53.1) 3 (2.7)	7 (6.2) 42 (37.2) 56 (49.6) 8 (7.0)	0.59
Duration of surgery in minutes	149.9 ± 65.8	163.3 ± 105.5	0.25

*variable implemented in propensity score matching

values as numbers and percentage or in means ± standard deviation

ASA - American Society of Anesthesiologists; BMI - Body Mass Index; COPD - Chronic Obstructive Pulmonary Disease; CNI - calcineurine inhibitor; EHS - European Hernia Society; MMF - Mycophenolat-Mofetil; IBD – inflammatory bowel disease, LT – liver transplantation; KT – kidney transplantation; OT – organ transplantation

**Table 7:** Comparison of early postoperative outcome in patients with and without immunosuppression after propensity score matching

	Immunosuppression (Group IS) *n* = 113	No Immunosuppression (Group nIS) *n* = 113	*p* value
Patients with postoperative complications	31 (27.4)	31 (27.4)	0.88
Patients with postoperative surgical complication	15 (13.3)	24 (21.2)	**0.02**
Typ of complications*			
SSI Seroma Bleeding/hematoma Paralytic Ileus Renal Cardiopulmonary Other	5 (4.4) 9 (8.0) 1 (0.9) 0 (0.0) 8 (7.1) 5 (4.4) 21 (18.6)	9 (8.0) 9 (8.0) 1 (0.9) 3 (2.7) 2 (1.8) 7 (6.2) 17 (15.0)	0.27 0.27 1.0 0.08 **0.05** 0.55 0.7
CDC Grade of complication			
≤IIIa ≥IIIb	20 (17.7) 12 (10.6)	23 (20.3) 8 (7.1)	0.32
Length of Hospital stay in days	9.7 ± 11.8	8.6 ± 10.4	0.46

*multiple selection possible

Values as numbers and percentage or in means ± standard deviation

SSI – surgical site infection; CDC – Clavien-Dindo Classification

## Discussion

This retrospective study aimed to investigate safety and long-term outcome of mesh-implantation for incisional hernia repair in immunosuppressed patients.

Variables associated with occurrence of postoperative complications in the overall cohort were common clinical parameters such as age and comorbidities (COPD, diabetes, nicotine consumption) [30–32]. However, in this cohort, BMI was not associated with postoperative complications. Length of surgery over 120 min and EHS width showed statistically significant impact on occurrence of postoperative complication, as has been reported in a recent meta-analysis and a large cohort study, respectively [27, 33]. In multivariate analysis of these parameters, age, COPD and duration of surgery showed statistical significance. A statistical impact of immunosuppression on postoperative complications was only observed in univariate analysis, but not in multivariate analysis.

We applied CART analysis to stratify variables for impact on postoperative complications. Interestingly, length of operation was associated with highest impact on occurrence of complications and here, longer operations in combination with preexisting pulmonary disease was relevant. This reflects routine clinical experience in surgery; anesthesia duration exceeding 2.5 h has been associated with a three-fold risk of postoperative pulmonary complications [34].

After propensity score matching, overall complications did not differ between patients with and without immunosuppression. This observation comes in line with recent cohort reports, but opposing findings also exist [19, 20, 35].

In this study, immunosuppressive therapy increased the risk of specific postoperative complications e.g. renal dysfunction when compared to the non-immunocompromised cohort. All of these patients had received organ transplantation and this collective is known to suffer of various comorbidities while at the same time those comorbidities are risk factors for complications after abdominal and other surgical interventions [9, 36–38]. In the cohort of immunosuppressed patients, elderly patients were associated with an elevated risk for complications after operation. However, no other comorbidity reached statistically significant impact, which might be explained due to relatively low numbers. Number of immunosuppressive agents above two and duration of immunosuppressive status over four years revealed negative impact within this cohort and maintained statistical relevance in multivariate analysis. Correspondingly, CART analysis identified the amount of immunosuppressants as most relevant factor for postoperative complications. As mentioned before, effects of immunosuppression on surgical procedures are widely known as well as the immunosuppressive effect of surgery itself [16, 18, 39–41]. We did not find a previous study to report on the association of the number of administered immunosuppressants and complications, however, we did not assess dosage or trough levels. Recommendations of perioperative handling of immunotherapy remain inconclusive with only poor data and recent guidelines of the World Health Organization for SSI prevention therefore recommend no alteration in the perioperative setting [42]. Our data certainly support this statement.

However, as more than two simultaneously administered immunosuppressants resulted in a significant increase in postoperative complications, a potential benefit might be achieved for a subset of patients when the number of immunosuppressants is reduced in the perioperative setting rather than a dosage reduction. Of interest, combination of immunosuppressants have been observed to amplify their effects: caIcineurin inhibitors might increase immunosuppressive function of steroids and triple immunosuppressive therapy has been reported to affect cytokine profiles beyond the individual drug´s capacity [43, 44].

Still, the choice of substances that might be prone to be paused remains to be evaluated. In LT patients, the phenomenon of tolerance is known, potentially enabling the strategy of pausing the immunosuppression in the perioperative, elective setting. Especially elderly patients might benefit from this [45–47].

Cumulative mesh-infection rate after hernia repair is reported to be up to 8% and recurrence rate of hernia after using mesh is still up to 30% after 10 years [11, 48]. Recurrence rate in immunosuppressed patients in this study was 14% with a mean follow up of two years. Evaluation of hernia recurrence was therefore limited. Still, our data suggest no detriment for immunosuppressed patients, as estimated recurrence rates of more than 12% have been reported after 2 years [7]. Younger patients were associated with higher rate of recurrence in the group of immunocompromised patients, which is contradictory to other reports [49–51]. However, a study reporting on on a US database with over 30,000 patients found no age-dependency in ventral hernia repair using mesh and a study based on the Herniamed-Registry found less recurrences in patients > 80 years old [35, 52].

In this study, a laparoscopic approach for incisional hernia repair was associated with increased hernia recurrence in the cohort of immunosuppressed patients. In general, laparoscopic hernia repair has been shown to be equal to open approaches regarding postoperative outcome with benefits in pain, mobilization and length of hospital stay. However, it is mainly recommended in hernia size up to 10 cm maximum [11, 53, 54]. In our cohort, most immunosuppressed patients presented with herniae > 10 cm combined with higher classification in ASA-grading. Thus, the surgical approach might have been influenced by overall morbidity towards minimal-invasive technique, but with the concomitant elevated risk of hernia recurrence. The inferior outcome after laparoscopic hernia repair in our cohort therefore might reflect the complexity of patients´ medical history and should not be generalized.

Of importance, duration of surgery exceeding 120 min and intake of more than two immunosuppressant at time of surgery were associated with increased recurrence of hernia, highlighting the importance of these aspects in this cohort.

Certain limitations of this study have to be addressed. Given its retrospective, single-center character, multiple potential bias might occur. In the setting of a retrospective, exploratory analysis in a large cohort, some findings might be sole statistical phenomena and clinical experience and day-to-day care might not reflect these observation. However, propensity score matching allowed for correction between groups. Thus, certain findings from the overall should be interpreted with the findings from the propensity score matching.

As classification of “immunosuppressed” remains vague due to lack of a coherent definition, some aspects may have been overlooked. Of note, diabetes or an underlying oncological disease/history of disease is sometimes considered as immunosuppressive [55, 56]. Further, we did not assess dosage or serum levels of immunosuppression or reason for combination of immunosuppressants. While increased dosage might hamper e.g. wound healing, most immunosuppressants in this context are trough-level based with focus on rejection prophylaxis in an individualized manner. The clinical concepts or reasonings for multi-drug combinations were not assessed in this analysis.

CART was applied in this analysis. While it is mainly used in analysis of decision-making, it has been used to define and compare categorical variables for their proportion towards a certain outcome variable before [57, 58].

## Conclusion

In this study, incisional hernia repair using mesh proved safe in immunosuppressed patients without increased adverse surgical outcome, especially without increase of SSI. Number of immunosuppressants administered and a longer history of immunosuppression however may increase overall postoperative complications in a subset of patients. To the best of our knowledge, this is the first systematical evaluation of safety of mesh implantation for hernia surgery in immunosuppressed patients with important insight into its feasibility. Still, optimal handling and risks of immunosuppression in surgery and especially hernia repair using allogenic material remain obscure and require further research, preferably in randomized controlled trials.

## Electronic supplementary material

Below is the link to the electronic supplementary material.

Supplementary Material 1

## Abbreviations

ASA: American Society of Anesthesiologists
BMI: Body Mass Index
CI: Confidence Interval
COPD: Chronic obstructive pulmonary disease
EHS: European Hernia Society
HIV: Human Immunodeficiency Virus
HR: Hazard Ratio
IBD: Inflammatory Bowel Disease
IPOM: Intraperitoneal Onlay Mesh
KT: Kidney Transplantation
LT: Liver Transplantation
OT: Organ Transplantation
PRO: Patient reported outcome
SD: Standard Deviation
SSI: Surgical Site Infection
