# Supplementary material for: Safety and outcome of elective synthetic mesh repair for incisional ventral hernias in immunosuppressed patients – a retrospective propensity-score-matched analysis
Source: Hernia. 2025 Feb 24;29(1):106. doi: 10.1007/s10029-025-03273-3 (PMC11850561; doi:10.1007/s10029-025-03273-3)
Supplement: Supplementary file 1 — Supplementary Material 1 [file 10029_2025_3273_MOESM1_ESM.docx]

| **variable** | **Patients with postoperative complications (%)** | **p value** |
| --- | --- | --- |
| Age  <60 years  ≥60 years | 59 (16.7)  107 (28.5) | **<0.001** |
| BMI  <30 kg/m²  >30 kg/m² | 91 (21.1)  58 (23.4) | 0.491 |
| ASA  1/2  3/4 | 65 (18.8)  76 (30.5) | **<0.001** |
| Comorbidities  COPD  Active nicotine consumption  Diabetes  Oncologic disease  Immunosuppression | 29 (38.7)  18 (16.1)  39 (29.5)  66 (24.7)  119 (21.0) | **<0.001**  0.066  **0.040**  0.340  **0.036** |
| EHS  Midline (M1-5)  Lateral (L1-4) | 126 (22.8)  40 (22.7) | 0.987 |
| EHS  W1  W2  W3 | 9 (11.1)  68 (20.7)  89 (27.8) | **0.003** |
| Surgical approach  open  laparoscopic | 109 (24.2)  57 (20.5) | 0.252 |
| Duration of surgery  <120 min  >120min | 45 (14.5)  121 (29.0) | **<0.001** |

**Supplement Table 1** **Univariate Analysis of impact of clinical variables associated with**  **postoperative complications in the overall cohort**

Values as numbers and percentage

ASA American Society of Anesthesiologists, BMI Body Mass Index, COPD Chronic Obstructive Pulmonary Disease; EHS European Hernia Society

| **variable** | **n** | **Hazard Ratio** | **95% CI** | | | **p value** |
| --- | --- | --- | --- | --- | --- | --- |
|  |  |  | **lower** | | **upper** |  |
| Age (<60* vs. ≥60 years) | 594 | 0.507 | 0.332 | 0.776 | | **0.002** |
| ASA (1/2* vs 3/4) | 594 | 0.799 | 0.521 | 1.225 | | 0.303 |
| COPD (yes* vs no) | 594 | 1.871 | 1.054 | 3.322 | | **0.032** |
| Diabetes (yes* vs no) | 594 | 1.305 | 0.795 | 2.143 | | 0.293 |
| Immunosuppression (yes* vs no) | 594 | 0.754 | 0.466 | 1.219 | | 0.249 |
| EHS (W1*) vs.  W2  W3 | 594 | 0.502  0.770 | 0.220  0.507 | 1.147  1.117 | | 0.102  0.22 |
| Duration of surgery (<120* vs. >120 min) | 594 | 0.457 | 0.293 | 0.715 | | **<0.001** |

**Supplement Table 2** **Multivariate logistic regression of parameters associated with postoperative complications in patients undergoing incision hernia repair**

*Reference

Values as numbers

CI - Confidence interval;
